# Combining plasma Epstein-Barr virus DNA and nodal maximal standard uptake values of ^18^F-fluoro-2-deoxy-D-glucose positron emission tomography improved prognostic stratification to predict distant metastasis for locoregionally advanced nasopharyngeal carcinoma

**DOI:** 10.18632/oncotarget.5699

**Published:** 2015-10-13

**Authors:** Wen-Hui Chen, Lin-Quan Tang, Lu Zhang, Qiu-Yan Chen, Shan-Shan Guo, Li-Ting Liu, Wei Fan, Xu Zhang, Ling Guo, Chong Zhao, Ka-Jia Cao, Chao-Nan Qian, Xiang Guo, Dan Xie, Mu-Sheng Zeng, Hai-Qiang Mai

**Affiliations:** ^1^ Sun Yat-sen University Cancer Center; State Key Laboratory of Oncology in South China; Collaborative Innovation Center for Cancer Medicine, Guangzhou 510060, China; ^2^ Department of Nasopharyngeal Carcinoma, Sun Yat-sen University Cancer Center, Guangzhou 510060, China; ^3^ Department of Nuclear Medicine, Sun Yat-sen University Cancer Center, Guangzhou 510060, China

**Keywords:** nasopharyngeal carcinoma, EBV DNA, SUVmax, survival

## Abstract

**Background:**

This study aimed to evaluate the value of combining the nodal maximal standard uptake values (SUVmax) of ^18^ F-fluoro-2-deoxy-D-glucose positron emission tomography with Epstein-Barr virus DNA(EBV DNA) levels to predict distant metastasis for nasopharyngeal carcinoma (NPC) patients

**Patients and Methods:**

Eight hundred seventy-four patients with stage III-IVa-b NPC were evaluated for the effects of combining SUVmax and EBV DNA levels on distant metastasis-free survival (DMFS), disease-free survival (DFS) and overall survival (OS).

**Results:**

The optimal cutoff value was 6,220 copies/mL for EBV DNA and 7.5 for SUVmax-N. Patients with lower EBV DNA levels or SUVmax-N had a significantly better 3-year DMFS, DFS, and OS. Patients were divided into four groups based on EBV DNA and SUVmax-N, as follows: low EBV DNA and low SUVmax-N (LL), low EBV DNA and high SUVmax-N (LH), high EBV DNA and low SUVmax-N (HL), and high EBV DNA and high SUVmax-N (HH). There were significant differences between the four mentioned groups in 3-year DMFS: 95.7%, 92.2%, 92.3%, and 80.1%, respectively (P^trend^ < 0.001). When looking at the disease stage, the 3-year DMFS in group LL, LH, HL, HH were 94.2%, 92.9%, 95.0%, and 81.1%, respectively, in stage III patients (P^trend^ < 0.001) and 92.7%, 87.2%, 86.3%, and 77.0% in stage IVa–b patients (P^trend^ = 0.026).

**Conclusion:**

Pretreatment EBV DNA and SUVmax of neck lymph nodes were independent prognostic factors for distant metastasis in NPC patients. Combining EBV DNA and SUVmax-N led to an improved risk stratification for distant metastasis in advanced-stage disease.

## INTRODUCTION

Nasopharyngeal carcinoma (NPC) is different from other head and neck malignancies terms of its pathology, epidemiology, and treatment outcome [1]. Currently, Intensity-modulated radiotherapy (IMRT), which exerts superior locoregional control [2, 3], has gradually replaced 2D-CRT as the primary means of radiotherapy in clinical practice. However, distant metastasis is still the greatest challenge for NPC patients. The American Joint Committee on Cancer (AJCC) TNM classification, based on anatomical information, is the most commonly used staging system and the benchmark for establishing treatment regimens for NPC patients. Currently, concurrent chemoradiotherapy with or without adjuvant chemotherapy is the primary regimen for patients with locoregionally advanced NPC. Nevertheless, approximately 20–30% of advanced NPC patients still develop distant failures within 3 years after completing treatment [4, 5].

Although many attempts have been made to reduce the rates of distant metastasis such as adding neoadjuvant or adjuvant chemotherapy, recent published data have indicated no significant benefit of neoadjuvant/adjuvant chemotherapy on the reduction of distant failure in patients with advanced NPC [6–9]. Another approach to reducing distant failure is selecting patients with occult distant metastases in initial staging. Our previous study demonstrated that ^18^FDG PET/CT was useful in revealing occult distant metastases, and combining the pre-treatment plasma EBV DNA level can guide the application of ^18^FDG PET/CT more appropriately [10]. The value of SUV_max_ for the tumor, as derived from ^18^FDG PET or ^18^FDG PET/CT scans has been found to be extremely valuable in predicting prognosis in NPC patients [11–13], and it was considered a potential biomarker to guide individualized treatment for NPC patients.

Recently, pre-treatment plasma Epstein-Barr virus (EBV) DNA levels were clinically employed for NPC diagnosis, risk stratification, monitoring and prognosis [14–18]. EBV DNA levels are considered the most attractive of the potential biomarkers that complement TNM classification in NPC [19]. Given the biological heterogeneity of cancer, the present staging system, even in combination with plasma EBV DNA levels, remains inadequate for predicting NPC prognosis. Although the independent prognostic value of SUVmax and plasma EBV DNA levels for NPC patients has been reported in previous studies, little is known regarding the value of combining the SUVmax and plasma EBV DNA to predict distant metastasis for NPC patients in clinical practice. Therefore, we hypothesized that a combination of SUVmax and plasma EBV DNA levels would improve prognostic stratification to predict distant metastasis for locoregionally advanced nasopharyngeal carcinoma. The purpose of this study was to evaluate the value of combining SUVmax and plasma EBV DNA for predicting NPC patient survival.

## RESULTS

### Patient characteristics and association with clinical variables

**Figure 1:**
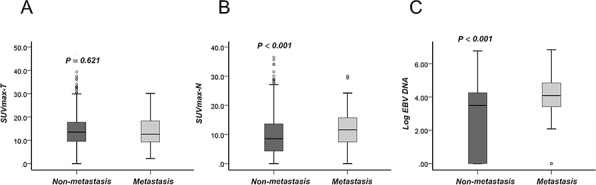
SUVmax-T, SUVmax-N and Log(EBV DNA) are expressed as the median and 5%–95% percentile in patients with/without distant metastasis **A.** SUVmax-T; **B.** SUVmax-N; **C.** Log(EBV DNA).

The characteristics of the 874 NPC patients are listed in Table 1. The median follow-up time was 37.3 months (interquartile range [IQR]: 30.5–47.9). The median SUVmax-N values in patients with (*n* = 88) and without (*n* = 786) distant metastasis were 11.6 (IQR: 7.28–15.8) and 8.5 (IQR: 4.28–13.6), respectively (*P* < 0.001, Fig. 1B)). However, there was no significant difference in the SUVmax-T value between the patients with and without distant metastasis (*P* = 0.621, Fig. 1A). The median EBV DNA levels were 12,150 (IQR: 2,600–69,825) copies/ml and 3,640 (IQR: 0–18,025) copies/ml in patients with and without distant metastasis, respectively (*P* < 0.001, Fig. 1C). When examined as continuous variables, SUVmax-T (*r*_s_ = 0.15, *P* = 0.002) and SUVmax-N (*r*_s_ = 0.433, *P* < 0.001) were positively correlated with EBV DNA. In addition, both the SUVmax-N and EBV DNA tertiles were significantly correlated with T stage (*P* < 0.01) and N stage (*P* < 0.001), but the SUVmax-T tertiles were significantly correlated only with the T stage (*P* < 0.001). In total, 45 patients developed locoregional relapse, 88 patients had distant metastases, and 58 were dead at the last follow-up.

**Table 1:** Patient Demographics and Clinical Characteristics

Number of patients (tertile of SUVmax-T, SUVmax-N and EBV DNA, *n* = 874)												
Characteristic	SUVmax-T			*P*	SUVmax-N			*P*	EBV DNA, copies/mL			*P*
	<10.7	10.7–16.1	≥16.1		<5.7	5.7–12.3	≥12.3		<629	629–9,820	≥9,820	
Age, years												
Median	48	46	48		46	48	47		46	47	48	
Sex				0.527				0.380				0.894
Female	63	72	61		61	74	61		64	64	68	
Male	229	220	229		232	220	226		227	228	223	
Histology, WHO type				0.873				0.019				0.318
II	8	7	9		2	13	9		5	11	8	
III	284	285	281		291	281	278		286	281	283	
Clinical stage				0.242				<0.001				<0.001
III	196	178	179		225	179	149		235	190	128	
IVa-b	96	114	111		68	115	138		56	102	163	
Tumor stage				<0.001				0.009				<0.001
T1	19	6	5		8	8	14		13	6	11	
T2	55	29	5		20	39	30		20	33	36	
T3	167	177	189		203	169	161		214	177	142	
T4	51	80	91		62	78	82		44	76	102	
Node stage				0.192				<0.001				<0.001
N0	34	41	33		94	11	3		60	33	15	
N1	84	96	113		124	102	67		134	103	56	
N2	124	112	107		65	133	145		83	127	133	
N3	50	43	37		10	48	72		14	29	87	
Treatment				0.512				<0.001				<0.001
RT	17	23	16		26	16	14		27	18	11	
CCRT	103	84	93		125	84	71		133	93	54	
NACT+CCRT	164	180	176		133	190	197		130	171	219	
CCRT+AC	8	5	5		9	4	5		1	10	7	
Radiotherapy technique				0.525				0.823				0.168
2DRT/3DCRT	73	70	81		72	75	77		70	68	86	
IMRT	219	222	209		221	219	210		221	224	205	
VCA-IgA				0.210				0.020				<0.001
<1:80	84	89	70		99	73	71		108	82	53	
≥1:80	208	203	220		194	221	216		183	210	238	
EA-IgA				0.156				<0.001				<0.001
<1:10	126	129	107		150	102	110		153	119	90	
≥1:10	166	163	183		143	192	177		138	173	201	
Smoking				0.302				0.132				0.09
No	170	188	180		191	183	164		194	172	172	
Yes	122	104	110		102	111	123		97	120	119	
Family history of NPC				0.855				0.876				0.102
No	258	262	257		261	263	253		266	261	250	
Yes	34	30	33		32	31	34		25	31	41	

Abbreviations: 2DRT = two-dimensional radiotherapy; 3DCRT = three-dimensional conformal radiotherapy; IMRT = intensity-modulated radiotherapy; VCA = viral capsid antigen; EA = early antigen; NPC = nasopharyngeal carcinoma follow-up; RT = radiation alone; CCRT = concurrent chemoradiotherapy; NACT + CCRT = neoadjuvant chemotherapy + concurrent chemoradiotherapy; CCRT + AC =concurrent chemoradiotherapy + adjuvant chemotherapy

### HRs and 95% CIs comparing SUVmax-N and EBV DNA tertiles

**Figure 2:**
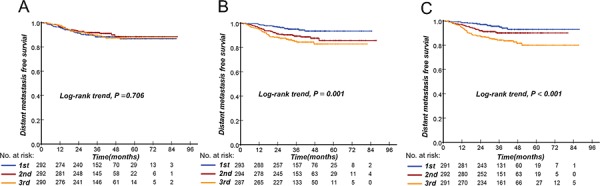
Upper SUVmax-T **A.** SUVmax-N **B.** and EBV DNA **C.** tertiles are associated with distant metastasis-free survival. *P* value for variables as determined by log-rank significance tests.

The cumulative DMFS probabilities for NPC patients indicated that EBV DNA tertiles are superior to predict distant metastasis compared with SUVmax-N tertiles (Fig. 2). The ROC curve analysis continued to demonstrate that the plasma EBV DNA and SUVmax-N have better ability to predict distant metatassis compared with SUVmax-T, with area under the curve (AUC) values of 0.661, 0.615 and 0.529 for EBV DNA, SUVmax-N and SUVmax-T, respectively. The Cox regression analysis indicated that both SUVmax-N and EBV DNA were associated with DMFS (TNM stage-adjusted hazard ratio: 2.73, 95% confidence interval [CI]: 1.50 to 4.99 for top SUVmax-N tertiles; TNM stage-adjusted hazard ratio: 2.98, 95% CI: 1.60 to 5.55 for top EBV DNA tertiles; both compared with the bottom tertiles). Linear associations were observed for SUVmax-N tertiles 2 to 3 and EBV DNA tertiles 2 to 3. After adjusting for age and other risk factors (Tables 2 and 3), the upper tertiles of both biomarkers remained associated with DMFS (P for trend < 0.01). The hazard ratios were 2.72 (95% CI: 1.48 to 5.00) for the upper SUVmax-N tertiles and 2.98 (95% CI, 1.57 to 5.65) for the upper EBV DNA tertiles.

**Table 2:** HRs for Distant Metastases-Free, Disease-Free and Overall Survival by SUVmax-N Tertiles for the Entire Population

Endpoints	Tertile of SUVmax-N, *n* = 874			*P* (trend)
	1	2	3	
	<5.7 (Bottom)	5.7–12.3	≥12.3	
**DMFS**				
TNM stage-adjusted^#^	1.00	2.04 (1.10–3.79)	2.73 (1.50–4.99)	0.005
Plus risk factors*	1.00	1.99 (1.06–3.75)	2.72 (1.48–5.00)	0.005
Plus EBV DNA^&^	1.00	1.75 (0.92–3.32)	2.06 (1.09–3.91)	0.084
**DFS**				
TNM stage-adjusted^#^	1.00	1.93 (1.16–3.19)	2.68 (1.65–4.37)	<0.001
Plus risk factors*	1.00	1.81 (1.08–3.02)	2.62 (1.60–4.29)	0.001
Plus EBV DNA^&^	1.00	1.70 (1.01–2.86)	2.31 (1.37–8.88)	0.007
**OS**				
TNM stage-adjusted^#^	1.00	2.26 (0.99–5.17)	3.70 (1.67–8.17)	0.004
Plus risk factors*	1.00	2.26 (0.97–5.29)	3.73 (1.66–8.35)	0.004
Plus EBV DNA^&^	1.00	2.14 (0.91–5.03)	3.28 (1.41–7.63)	0.019

Abbreviations: The values represent hazard ratios (95% CI).

# Obtained from Cox proportional hazard regression models adjusted for TNM stage (IV vs. III).

* Obtained from Cox proportional hazard regression models adjusted for age (≥47 years vs. < 47 years), sex (male vs. female), WHO pathological type (undifferentiated non-keratinizing vs. differentiated non-keratinizing), chemoradiotherapy (yes vs. no), radiation technique (IMRT vs. 3D-CRT/2D-CRT), VCA (≥ 1:80 vs. < 1:80), EA (≥ 1:10 vs. < 1:10), smoking status (yes vs. no), and family history of NPC (yes vs. no). The lowest tertile of each biomarker served as the reference category for the hazard ratios. P values were obtained from models, which were used to assess linear trends.

& Adjusted for all the above variables and EBV DNA

**Table 3:** HRs for Distant Metastases-Free, Disease-Free and Overall Survival by EBV DNA Tertiles for the Entire Population

Endpoints	Tertile of EBV DNA, copies/mL, *n* = 874			*P* (trend)
	1	2	3	
	<629	629–9,820	≥9,820	
**DMFS**				
TNM stage-adjusted^#^	1.00	1.78 (0.93–3.41)	2.98 (1.60–5.55)	0.002
Plus risk factors*	1.00	1.75 (0.91–3.39)	2.98 (1.57–5.65)	0.002
Plus SUVmax-N^&^	1.00	1.54 (0.79–2.99)	2.33 (1.19–4.57)	0.037
**DFS**				
TNM stage-adjusted^#^	1.00	1.14 (0.69–1.88)	1.98 (1.24–3.15)	0.004
Plus risk factors*	1.00	1.08 (0.65–1.80)	1.84 (1.14–2.97)	0.011
Plus SUVmax-N^&^	1.00	0.92 (0.55–1.54)	1.37 (0.82–2.27)	0.169
**OS**				
TNM stage-adjusted^#^	1.00	1.49 (0.68–3.29)	2.28 (1.08–4.81)	0.070
Plus risk factors*	1.00	1.39 (0.62–3.09)	2.15 (1.0–4.65)	0.111
Plus SUVmax-N^&^	1.00	1.12 (0.50–2.53)	1.44 (0.64–3.23)	0.340

Abbreviations: The values represent hazard ratios (95% CI).

# Obtained from Cox proportional hazard regression models adjusted for TNM stage (IV vs. III).

* Obtained from Cox proportional hazard regression models adjusted for age (≥47 years vs. < 47 years), sex (male vs. female), WHO pathological type (undifferentiated non-keratinizing vs. differentiated non-keratinizing), chemoradiotherapy (yes vs. no), radiation technique (IMRT vs. 3D-CRT/2D-CRT), VCA (≥ 1:80 vs. < 1:80), EA (≥ 1:10 vs. < 1:10), smoking status (yes vs. no), and family history of NPC (yes vs. no). The lowest tertile of each biomarker served as the reference category for the hazard ratios. *P* values were obtained from models, which were used to assess linear trends.

& Adjusted for all the above variables and SUVmax-N.

We further adjusted SUVmax-N for EBV DNA in a Cox model. The hazard ratio comparing the top and bottom SUVmax-N tertiles was slightly attenuated to 2.06 (95% CI: 1.09 to 3.91), although the trend across tertiles reached a marginal significant difference (P for trend = 0.084) (Table 2). Similarly, in a Cox model that adjusted EBV DNA for SUVmax-N tertiles, the hazard ratio comparing the top and bottom EBV DNA tertiles was also mildly attenuated to 2.33 (95% CI: 1.19 to 4.57), but the trend across tertiles remained significant (P for trend = 0.037) (Table 3). A similar finding was observed for DFS and OS regardless of HR adjusted for TNM stage or other risk factors. Multiplicative interactions between SUVmax-N tertiles and EBV DNA categories with regard to DMFS, DFS and OS were not observed; the *P* values for these interactions were 0.370, 0.113, and 0.654, respectively.

### Association of elevated SUVmax-N and plasma EBV DNA levels with DMFS, DFS, and OS

**Figure 3:**
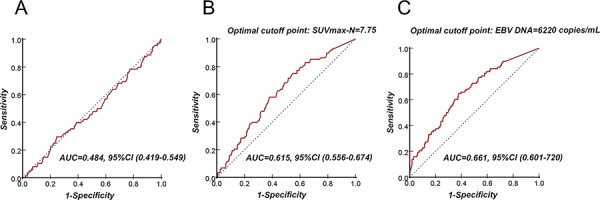
Receiver operating characteristic (ROC) curve analysis of the cutoff point for distant metastasis–free survival (DMFS): SUVmax-T **A.** SUVmax-N **B.** and EBV DNA **C.** At each point, the sensitivity and specificity for the outcome being studied were plotted to generate an ROC curve. The optimal cutoff points of SUVmax-N and EBV DNA for DMFS were 7.75 and 6,220 copies/mL, respectively. Receiver operating characteristic (ROC) curve analysis presented the different AUC value of SUVmax-T, SUVmax-N and EBV DNA for predicting distant metastasis–free survival.

**Figure 4:**
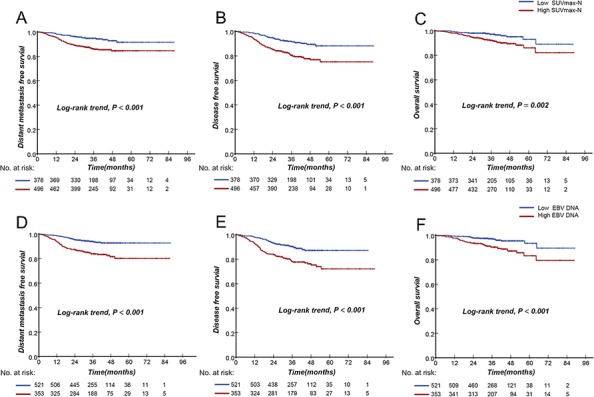
Kaplan-Meier analysis of the survival of the subgroup of patients with a high SUVmax-N or EBV DNA compared to the subgroup with low SUVmax-N or EBV DNA DMFS **A.** DFS **B.** and overall survival **C.** of SUVmax-N; DMFS **D.** DFS **E.** and overall survival **F.** of EBV DNA; Low SUVmax-N denotes a low SUVmax-N of <7.75; High SUVmax-N denotes a high SUVmax-N of ≥7.75; Low DNA denotes a low EBV DNA level of <6220 copies/mL; High DNA denotes a high EBV DNA level of ≥6220 copies/mL.

The optimal cutoff point for predicting distant metastasis was determined by ROC curve analysis. As shown in Fig. 3, the optimal cutoff point of SUVmax-N and EBV DNA for DMFS was 7.75 and 6,220 copies/mL, respectively. Compared with the patients with SUVmax-N < 7.75, patients with SUVmax-N ≥ 7.5 had a shorter three-year DMFS (85.9%, 95%CI (82.6–89.2)) vs. (94.9%, 95%CI (92.5–97.3, *P* < 0.001)). For the patients with EBV DNA levels ≥ 6,220 copies/mL compared with those displaying EBV DNA levels < 6,220 copies/mL, the 3-year DMFS was 83.9% (95%CI,79.9%-87.8%) and 94.1% (95%CI,91.9%-96.3%), respectively (*P* < 0.001). We noted similar results for DFS and OS (Fig. 4).

### Prognostic value of integrating plasma EBV DNA and SUVmax-N levels

**Figure 5:**
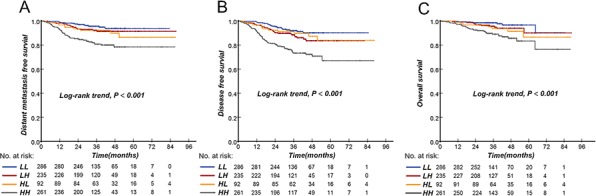
Kaplan-Meier curves of distant metastasis-free survival, disease-free survival, and overall survival according to the combination of pretreatment EBV DNA and SUVmax-N in NPC patients Distant metastasis-free survival **A.** disease-free survival **B.** and overall survival **C.** for 874 patients. LL, <6,220 copies/mL EBV DNA and <7.75 SUVmax-N; LH, <6,220 copies/mL EBV DNA and ≥7.75 SUVmax-N; HL, ≥6,220 copies/mL EBV DNA and <7.75 SUVmax-N; HH, ≥6,220 copies/mL EBV DNA and ≥7.75 SUVmax-N.

The advanced-stage NPC patients were divided into 4 subgroups: low EBV DNA and low SUVmax-N (LL), low EBV DNA and high SUVmax-N (LH), high EBV DNA and low SUVmax-N (HL), and high EBV DNA and high SUVmax-N (HH). Interestingly, there was a significant difference in DMFS, DFS, and OS between the four subgroups (P^trend^ < 0.001, Fig. 5). Reduced DMFS, DFS, and OS were significantly associated with an increased value of both EBV DNA and SUVmax-N. Increased DMFS, DFS, and OS were significantly associated with low levels of both biomarkers. Of note, patients with high EBV DNA and low SUVmax-N displayed increased event rates during follow-up compared with patients who had low EBV DNA and SUVmax-N values.

The 3-year DMFS for stages III and IVa-b was 91.9% and 86.1%, respectively. In subgroup analysis of stage III patients, however, we found that the 3-year DMFS was 94.2%, 92.9%, 95.0%, and 81.1% in groups LL, LH, HL and HH, respectively(P^trend^ < 0.001). For stage IVa-b patients, the 3-year DMFS for group LL, LH, HL and HH was 92.7%, 87.2%, 86.3%, and 77.0%, respectively (P^trend^ < 0.001, Table 4). These results indicated that combining EBV DNA and SUVmax-N would identify approximately 20% of stage III and 40% of stage IVa-b patients who have a high risk of developing distant metastasis. All analyses were repeated using DFS and OS as the end point, and the same conclusions as those for DMFS were obtained (data not shown).

**Table 4:** Log-Rank Test on DMFS for TNM Stages Split by EBVDNA and SUVmax-N Combination

TNM stage	No.of patients (%)	DMFS	
		Events (No.)	3- years' DMFS(%)
**III+IVa-b**	874		
Low DNA+Low SUVmax-N	286 (32.7)	13	95.7 (93.2–98.2)
Low DNA+High SUVmax-N	235 (26.9)	18	92.2 (88.7–95.7)
High DNA+Low SUVmax-N	92 (10.5)	9	92.3 (87.8–96.8)
High DNA+High SUVmax-N	261 (29.9)	48	80.1 (74.8–85.4)
**III**	553	43	91.9 (89.5–94.3)
Low DNA+Low SUVmax-N	229 (41.4)	10	94.2 (90.1–97.8)
Low DNA+High SUVmax-N	160 (28.9)	11	92.9 (89.1–96.9)
High DNA+Low SUVmax-N	44 (8.0)	2	95.0 (89.1–100)
High DNA+High SUVmax-N	120 (21.7)	20	81.1 (77.2–96.8)
**IVa-b**	321	45	86.1 (82.2–90.0)
Low DNA+Low SUVmax-N	57 (17.8)	3	92.7 (82.2–100)
Low DNA+High SUVmax-N	75 (23.4)	7	87.2 (77.2–96.8)
High DNA+Low SUVmax-N	48 (15.0)	7	86.3 (74.2–97.7)
High DNA+High SUVmax-N	141 (43.8)	28	77.0 (69.2–84.8)

Abbreviations: low DNA defined as <6,220 copies/mL EBV DNA; high DNA defined as ≥6,220 copies/mL EBV DNA; low SUVmax-N defined as<7.75 SUVmax-N; high SUVmax-N defined as ≥7.75 SUVmax-N; DFS = disease free survival; DMFS = distant metastasis free survival; OS = overall survival. P values compared for overall log-rank trend test. Events (No.) = the total number of events that occurred at the last follow-up. Values in parentheses indicate 95% confidence interval ranges

## DISCUSSION

The IMRT technique has been widely used in clinical practice, and, although local control has been greatly improved, distant metastasis is still the greatest challenge in the IMRT era, especially for locoregionally advanced disease. Therefore, predicting distant metastasis and stratifying patients according to the risk of distant metastasis is of great importance. To the best of our knowledge, this is the first large-scale study to examine the role of combining plasma EBV DNA levels and SUVmax values to predict distant metastasis. Our results confirmed that SUVmax-N, but not SUVmax-T, of ^18^F-FDG PET/CT and pretreatment EBV DNA levels were valuable prognosticators for predicting distant metastasis. Combined analysis of plasma EBV DNA and SUVmax-N provided relevant incremental prognostic information for NPC patients. We found that elevated EBV DNA and SUVmax-N levels, alone and in combination, are associated with reduced DMFS, DFS, and OS. Despite the positive correlation between EBV DNA and SUVmax-N, elevated levels of these biomarkers together were associated with reduced survival. The predictive value of distant metastasis for EBV DNA was superior to that of SUVmax-N, and the combination of these biomarkers has a better predictive ability without evidence of a multiplicative interaction.

Previous studies [14, 16–18, 20] have examined the association between EBV DNA and NPC prognosis, but its clinical utility has not been fully established. In addition, we have recently found that the pretreatment plasma EBV DNA level was an independent factor to predict distant metastasis for NPC patients at the initial stage and would guide the clinical application of PET/CT according to the risk of distant metastasis [10]. Notably, Chang et al. recently reported that the SUVmax of the primary tumor and neck lymph nodes were independent prognostic factors for DMFS in NPC patients treated with IMRT.

However, the predictive value of SUVmax in combination with plasma EBV DNA has not been assessed in NPC. Interestingly, our findings demonstrated that EBV DNA and SUVmax-N had an additive effect to predict distant metastasis and suggest a complementary role for them in risk prediction. We found that both SUVmax-N and EBV DNA tertiles were significantly correlated with the N stage but that the SUVmax-T tertile was significantly correlated only with the T stage. Moreover, The N stage has widely been demonstrated to be an independent factor to predict distant metastasis [9, 10, 21]. This can partially explain why combining SUVmax-N and EBV DNA will improve the stratification to predict distant metastasis for NPC patients, whereas there was little predictive value for SUVmax-T.

Recent advancements have been made in NPC patient classification and NPC molecular alterations, including microRNA signatures and the NPC-SVM classifier [22, 23]. However, these developments required expensive and complicated procedures, and rapid clinical implementation was difficult to achieve. To date, routine distant metastasis risk assessment of NPC patients still relies on traditional clinical stage and EB virus-associated blood tests. SUVmax-N values are established and easily obtained in clinical practice. Thus, the combination of plasma EBV DNA and SUVmax-N value is a very useful tool to identify patients with a high risk of developing distant metastasis.

The identification of factors predictive of distant metastasis in cancer patients is of great interest because such a result could allow therapy to be tailored to the characteristics of an individual patient. In a subgroup analysis of stage III patients, who had both a higher EBV DNA and SUVmax-N (EBV DNA ≥ 6,220 copies/mL and SUVmax-N ≥ 7.5), the 3-year DMFS (81.1%) was worse than in the other three groups (92.9–95.0%). This new classification system categorized approximately 80% of the stage III patients as having a low risk and 20% of the patients as having a high risk of developing distant metastasis within 3 years, and more aggressive systemic treatment could be considered for this subgroup of patients.

The 3-year DMFS in stage IVA to IVB patients with both a lower EBV DNA and SUVmax-N (EBV DNA < 6,220 copies/mL and SUVmax-N < 7.5) was 92.7%, which was better than that for the other three groups (77.0 to 87.2%) and suggested that a more aggressive treatment could be used in stage IVA to IVB patients, except in those with both a lower EBV DNA and SUVmax-N (EBV DNA < 6,220 copies/mL and SUVmax-N < 7.5).

Concurrent chemoradiotherapy (CCRT) with or without adjuvant chemotherapy is currently considered as the standard treatment regimens of locoregionally advanced nasopharyngeal carcinoma. Approximately 93% of the patients with stage III-IVa-b received platinum-based chemotherapy in this study, according to the principles of treatment for NPC patients at the study institute. It indicated that the current standard treatment therapy is still not sufficient to reduce the distant metastasis rate in high-risk advanced-stage NPC patients (approximately 20% in stage III patients and 40% in stage IVa-b patients, as identified by combining EBV DNA and SUVmax-N). Thus more aggressive systemic treatment that can reduce distant metastasis should be provided for this subgroup of patients, such as an adjusted dosage and course of chemotherapy, administration of an additional target agent (the EGFR inhibitor or bevacizumab), or the additional use of immunotherapy. Adoptive immunotherapy was considered a potential avenue for patients in the high-risk group, and we are initiating a phase II study using adoptively transferred tumor-infiltrating lymphocyte (TIL) immunotherapy following CCRT in patients with high risk of treatment failure [24].

There were several shortcomings of the current study. The major limitation is that evaluation of ^18^F-FDG PET uptake by SUV is semiquantitative, and it is difficult to compare SUV between cancer centers and patients. Second, the data obtained in this study were exclusively from one center in an endemic area, and the measurement of plasma EBV DNA still needs to be globally standardized. Although our cancer center treats a large number of NPC patients, these results need to be validated in other data sets. The third limitation is that the median follow-up time was 37.3 months. The patients remain closely followed, and we will report 5-year follow-up results when they are available. However, all of the previous studies demonstrated that higher a SUVmax was associated with poor prognosis. Nevertheless, our study is noteworthy because it is the largest-scale study to prove that combining plasma EBV DNA and SUVmax-N of ^18^FDG PET/CT improves prognostic stratification to predict distant metastasis for locoregionally advanced NPC patients.

In conclusion, our results demonstrated that both pretreatment EBV DNA and SUVmax of neck lymph nodes were independent prognostic factors for distant metastasis in NPC patients. Combining EBV DNA and SUVmax-N will enable better risk stratification and patient selection in future studies, thus allowing for future exploration of new aggressive systemic treatment to reduce distant metastasis.

## MATERIALS AND METHODS

This study combines the data from two cohorts of patients in two plasma EBV DNA and/or ^18^FDG PET/CT studies. The rationale for combining two cohorts of patients was to maximize the sample size used to evaluate the prognostic value of combining the SUVmax and plasma EBV DNA. The first cohort of patients comprised 416 stage III-IVa-b patients recruited in a previous prospective study to evaluate the benefit of ^18^FDG PET/CT test with the combination of plasma EBV DNA level [10]. The second cohort comprised 458 stage III-IVa-b patients recruited in another cohort study to assess the prognostic value of plasma EBV DNA and fibrinogen for NPC patients [25]. All these patients underwent the ^18^FDG PET/CT and plasma EBV DNA test within two weeks before treatment at the Sun Yat-sen University Cancer Center, Guangzhou, China. This study was approved by the Clinical Research Ethics Committee of the study institute, and the participants provided written informed consent before treatment.

### Clinical staging and treatment

All patients were restaged according to the seventh American Joint Committee on Cancer (AJCC) TNM staging manual [26]. The routine staging work-up included clinical examination of the head and neck region, magnetic resonance imaging scans from the suprasellar cistern to the collarbone, fiberoptic nasopharyngoscopy, chest radiography, abdominal sonography, and a whole-body bone scan or FDG PET/CT. In total, 224 (25.6%) patients were treated with conventional two-dimensional (2D) or three-dimensional (3D) conformal radiotherapy (CRT), and 650 (74.4%) patients were treated with IMRT. For the patients enrolled in this study, 56 (6.4%), 280 (32.0%), 520 (59.5%) and 18 (2.1%) were treated with radiation alone (RT), concurrent chemoradiotherapy (CCRT), neoadjuvant chemotherapy plus concurrent chemoradiotherapy (NACT+CCRT), and concurrent chemoradiotherapy plus adjuvant chemotherapy (CCRT+AC), respectively. Neoadjuvant or adjuvant chemotherapy consisting of cisplatin plus 5-fluorouracil or cisplatin plus taxane was administered every 3 weeks for 2 or 3 cycles [27]. All of chemotherapy regimen of CCRT was based on cisplatin every 3 weeks or weekly. The Kaplan–Meier survival analysis indicated that there is no statistically significantly difference for DMFS, DFS and OS among the subgroup patients treated with RT, CCRT, NACT+CCRT, and CCRT+AC, respectively (Supplemental Fig. 1). All patients were treated according to the principles of treatment for NPC patients at Sun Yat-sen University Cancer Center, Guangzhou, China.

### EBV DNA, VCA-IgA, and EA-IgA measurement

As previously described [18, 20, 28], patients' plasma EBV DNA concentrations were routinely measured by q-PCR before treatment. EBV-specific VCA/IgA antibodies and EBV-specific EA/IgA antibodies were assessed using a previously described immunoenzymatic assay [29].

### PET/CT imaging test

The serum glucose levels were measured in all of the NPC patients, who fasted for at least 6 hours prior to the PET/CT scans; participants with a fasting plasma glucose >200 mg/dl were excluded. PET/CT imaging was performed with a combination PET/CT scanner (Discovery ST 16, GE Healthcare) according to published guidelines for tumor imaging with PET/CT [30]. Helical CT was performed from the head to the proximal thigh prior to PET acquisition, according to a standardized protocol. PET/CT scans from the head to the proximal thigh were started at 45–60 min after the injection of 5.55 MBq/kg of FDG. The PET images were reconstructed with the use of CT data for attenuation correction with an ordered-subset expectation maximization iterative reconstruction algorithm [10].

We defined the SUVmax as the highest activity concentration per injected dose per body weight after correcting for radioactive decay. SUVmax-T was defined as the SUVmax at the primary tumor and the SUVmax-N as the highest SUVmax of neck nodes in this study. The images were analyzed by two qualified doctors experienced in PET-CT diagnosis (W. Fan and X. Zhang, Department of Nuclear Medicine, Sun Yat-sen University Cancer Center).

### Clinical outcome assessment and patient follow-up

Our primary endpoint was distant metastasis-free survival (DMFS), and our secondary endpoints were disease-free survival (DFS) and overall survival (OS). DMFS was determined from the date of treatment to the date of distant relapse or patient censoring at the date of the last follow-up. DFS was calculated from the date of treatment to the date of the first relapse at any site, death from any cause or the date of the last follow-up visit. OS was calculated from the date of treatment to the date of death from any cause or patient censoring at the date of the last follow-up. After the treatment was complete, the patients were evaluated at 3-month intervals for the first 3 years and every 6 months thereafter.

### Statistical analysis

Spearman rank correlation coefficients (r_s_) were calculated for continuous variables, and SUVmax-T, SUVmax-N and EBV DNA values were divided into tertiles. A chi-square test was applied to evaluate the correlation of SUVmax-T, SUVmax-N and EBV DNA tertiles with clinical outcome. The Kaplan–Meier method was used to estimate the cumulative survival plot in relation to the variables divided according to their tertiles. The survival among groups was compared using the log-rank test. Hazard ratios (HRs) and 95% CIs for EBV DNA and SUVmax-N tertiles were estimated using Cox proportional hazards regression. We first adjusted for TNM stage and then further adjusted for age (years), sex, pathological type, treatment allocation, VCA-IgA, EA-IgA, smoking (yes/no), and familial history of NPC (yes/no). To assess potentially confounding variables or effect mediation by other biomarkers, the models assessing the association of SUVmax with survival were further adjusted for EBV DNA and vice versa. We used ROC curve analysis to evaluate the ability of SUVmax-T, SUVmax-N and plasma EBV DNA to predict distant metastasis, and to select the optimal cutoff point of SUVmax and plasma EBV DNA for DMFS.

We then analyzed the combined association according to high or low SUVmax-N (optimal cutoff point: above or below 7.75) and high or low EBV DNA (optimal cutoff point: above or below 6,220 copies/mL). Finally, we performed statistical tests for interaction between SUVmax and EBV DNA tertiles using the TNM stage-adjusted Cox regression models. All reported probability values were two tailed, and *P* < 0.05 was considered significant. Statistical analyses were performed with SPSS 17.0.

## SUPPLEMENTARY FIGURE


