# Supplementary material for: Combining plasma Epstein-Barr virus DNA and nodal maximal standard uptake values of 18F-fluoro-2-deoxy-D-glucose positron emission tomography improved prognostic stratification to predict distant metastasis for locoregionally advanced nasopharyngeal carcinoma
Source: Oncotarget. 2015 Oct 13;6(35):38296–307. doi: 10.18632/oncotarget.5699 (PMC4742000; doi:10.18632/oncotarget.5699)
Supplement: Supplementary file 1 [file oncotarget-06-38296-s001.pdf]

SUPPLEMENTARY FIGURE

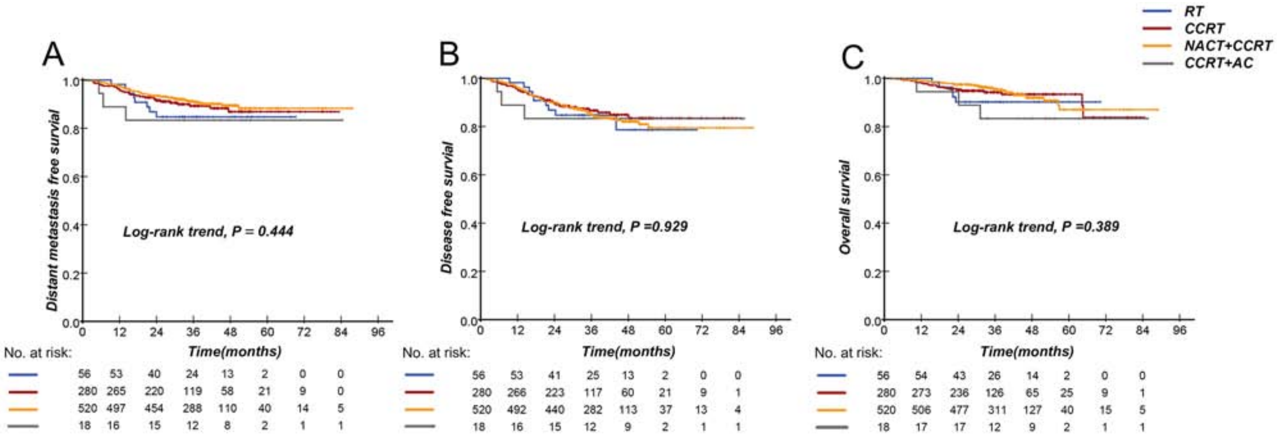

Supplementary Figure S1: Kaplan-Meier analysis of the survival of the subgroup patients treated with RT, CCRT, NACT + CCRT, and CCRT + AC, respectively. DMFS **A.** DFS **B.** and overall survival **C.** RT = radiation alone; CCRT = concurrent chemoradiotherapy; NACT + CCRT = neoadjuvant chemotherapy + concurrent chemoradiotherapy ; CCRT + AC = concurrent chemoradiotherapy + adjuvant chemotherapy
